# Uncorking Quantitative and Qualitative Seminal Traits in French-Alpine Bucks via Carbetocin Stimulation to Enhance Goat Production in Semi-Arid Environments

**DOI:** 10.3390/ani16193157

**Published:** 2026-10-08

**Authors:** Denia L. Vargas-García, Cayetano Navarrete-Molina, César A. Meza-Herrera, Ángeles De Santiago-Miramontes, Maurilio Solorio-Ochoa, Mayela Rodríguez-González, Irene Chavarría-Neri, María A. Sariñana-Navarrete, Martín Alfredo Legarreta-González, Pedro Antonio Robles-Trillo

**Affiliations:** 1 Graduate Program—Agricultural and Livestock Sciences, Laguna Unit, Antonio Narro Agrarian Autonomous University, Torreon 27054, Coahuila, Mexico; 2 Graduate Program—Natural Resources and Environment in Arid Lands, Regional Universitary Unit on Arid Lands, Chapingo Autonomous University, Bermejillo 35230, Durango, Mexico; 3 Division of Health, Biological and Environmental Sciences, Open and Distance University of Mexico, Ciudad de Mexico 03330, Mexico; 4 Department of Sustainable and Protected Agriculture, Technological University of Rodeo, Rodeo 35760, Durango, Mexico; 5 School of Engineering and Sciences, Tecnologico de Monterrey, Monterrey 64700, Nuevo Leon, Mexico

**Keywords:** male reproduction, oxytocin analogue, semen collection, spermatic flow, semen quality tracking, arid lands

## Abstract

Optimizing semen collection is critical for maximizing genetic advancement and reproductive efficiency in livestock. While exogenous hormones can enhance ejaculate quantity and quality, short windows of biological activity often limit their efficacy. This study was conducted in northern-arid Mexico (25° N, 103 W) to evaluate the comparative effects of oxytocin (OXYT) and carbetocin (CARB) administration on semen quality in French-Alpine bucks. Males (n = 12) were allocated into three groups and received intravenous treatments 10 min prior to artificial vagina collection: (1) OXYT (10 UI oxytocin buck^−1^), (2) CARB (0.2 mg 100 kg^−1^ LW^−1^), and (3) CONT (1 mL saline buck^−1^). The same bucks received all three treatments separately. CARB outperformed both groups (*p* ≤ 0.05), improving all evaluated parameters (*p* ≤ 0.05): ejaculated volume (i.e., increased by 103%), sperm concentration, mass and progressive motility, total sperm count, viability, normal morphology, plasma membrane functionality, acrosome integrity, and chromatin dispersion. Crucially, CARB doubled (*p* ≤ 0.05) the available projected or theoretical number of doses for intravaginal artificial insemination (POTNDIAI), followed by the OXYT and CONT groups, with 11.14 POTNDIAI each. Therefore, CARB emerges as a highly effective, accessible pharmacological strategy for semen collection protocols, substantially enhancing sperm yield and structural quality in breeding bucks. Ultimately, by bridging advanced reproductive physiology with practical smallholder farming, this methodological optimization provides a viable strategy to enhance reproductive efficiency and long-term sustainability in small ruminant production systems within arid environments.

## Simple Summary

The use of exogenous oxytocin-like hormones to enhance semen quality in breeding bucks remains understudied. Carbetocin, a synthetic oxytocin analogue, offers a promising pharmacological alternative due to its tenfold greater metabolic stability. This study evaluated the effects of pre-collection oxytocin and carbetocin administration on the seminal traits of French-Alpine bucks. Intravenous administration of carbetocin ten minutes prior to semen collection significantly enhanced all quantitative and qualitative traits, including volume of the ejaculate, sperm concentration, progressive motility, structural membrane integrity, and total viable sperm. Moreover, carbetocin doubled the number of potential doses available for artificial insemination per ejaculate. These findings position carbetocin as a highly efficient, practical tool to optimize semen collection protocols, maximize the output of superior studs, and support genetic conservation programs in goat production.

## 1. Introduction

In goat production, artificial insemination (AI) is a decisive tool for genetic improvement and increasing productivity [1,2]. In dairy production systems, the French-Alpine breed stands out due to its remarkable adaptability and high productive performance [3,4]. However, the efficiency of these programs directly depends on obtaining ejaculates with optimal quality parameters to ensure successful cryopreservation [5,6]. Semen collection via an artificial vagina (AV) in trained bucks is the preferred standard technique [7,8], because it accurately simulates the physiological conditions of natural mating. Consequently, this method reduces systemic stress responses compared to electroejaculation [9,10,11] and provides greater sperm structural stability during the post-thaw process [10,12]. To optimize ejaculate quality, the exogenous use of oxytocin (OXYT) has been investigated across various species [2,13,14,15,16,17], as this hypothalamic neuropeptide regulates sexual activity and ejaculatory mechanics in males [18]. Its administration prior to collection increases sperm concentration (SC) and total volume of the ejaculate (VE) in ruminants [18,19], by inducing an efficient emptying of epididymal sperm reserves and stimulating the secretory activity of accessory sex glands [20,21]. Nevertheless, the magnitude of these effects varies depending on the dose, administration route, and time elapsed before sexual stimulation [18,22,23].

The primary pharmacological limitation of OXYT is its short plasma half-life, estimated at approximately 2 to 3 min in small ruminants (SR) [22,24]. This rapid degradation demands precise timing to avoid compromising the collection protocol [23,25]. As a pharmacological alternative, CARB —a synthetic OXYT analogue with superior stability and up to a ten-fold longer duration of action [26,27]—offers greater technical flexibility [26,28]. While successfully utilized in veterinary medicine to stimulate uterine contractility and milk ejection [27,29,30], its application in males suggests the capacity to induce sustained contractions within the sperm transport system [26,31]. Furthermore, calculating accurate AI doses requires precise evaluation of SC [32,33,34]. Although rapid automated systems (e.g., computer-assisted semen analysis [CASA]) and flow cytometry are available, they are not yet widely used [35,36], especially under marginal production schemes. Besides, some factors, such as extender opalescence [37,38,39,40] and cell movement during analysis [41,42,43], may alter the results. Consequently, conventional microscopic counting chambers remain the most reliable reference method [36,44].

Optimizing semen collection under strict animal welfare standards is vital for high-merit sires [24,45,46,47], yet comparative studies between OXYT and CARB in French-Alpine bucks are lacking. This knowledge gap is critical for northern-arid Mexico, specifically in the Comarca Lagunera (C.L.), which is a highly specialized goat dairy cluster. In fact, although the C.L. holds only 2.8% of the national goat inventory (~8 million heads), it generates 36.2% (63 million L) of the national goat milk, predominantly based on a diurnal grazing system [48,49,50,51]. Given the intense genetic selection pressure on the foundational French-Alpine breed in this highly efficient region, improving fresh semen collection protocols is imperative. Building on these findings, we hypothesized that the strategic administration of CARB will optimize ejaculate characteristics and microscopic seminal quality traits, compared to OXYT, in French-Alpine bucks in northern Mexico (25° N). Consequently, the analytical matrix of this trial was developed to address this critical scientific inquiry.

## 2. Materials and Methods

All experimental methods, procedures, and animal management protocols were conducted in strict compliance with international and national guidelines for the ethical use, care, and welfare of research animals [52,53]. The study aligned with the Mexican Official Standard NOM-062-ZOO-1999 [54] and was formally approved by the Institutional Animal Care and Use Committee under reference number UAAAN-UL-425502002-2944.

### 2.1. Location & Environmental Conditions

The experimental period spanned from July 2025 to June 2026. The trial was performed at the Goat Academic Experimental Station, Department of Animal Production at the Universidad Autónoma Agraria Antonio Narro—Unidad Laguna. This facility is located in north-central Mexico within the Comarca Lagunera region (25°32′ N, 103°27′ W), a semi-arid ecosystem situated at an average altitude of 1120 m above sea level. The regional climate is characterized by an annual mean temperature of 23.8 °C (ranging from extreme minimums of −5 °C to maximums of 45 °C), a mean annual precipitation of 230 mm, and an average relative humidity of 22.55% (range: 12% to 61%). Photoperiod variations in the study area range from 13 h 41 min during the summer solstice (June) to 10 h 19 min during the winter solstice (December) [55,56].

### 2.2. Experimental Animals, Management, and Treatments

This study used French-Alpine clinically healthy bucks (n = 12), with a mean age of 3.6 ± 0.5 years, an average live weight (LW) of 62.5 ± 1.5 kg, and a mean body condition score (BCS) of 3.2 ± 0.4 units, determined according to the scale (i.e., range 1–4) described by Ponce-Covarrubias et al. [57]. All bucks were previously trained for semen collection using an AV and a dummy mount. Animals were housed individually in 6 m^2^ pens (3 m^2^ concrete floor and 3 m^2^ shaded area). The daily diet consisted of 750 g of alfalfa hay and 250 g of commercial concentrate (14% crude protein) offered twice daily (08:00 and 16:00 h), meeting the nutritional requirements recommended by the National Research Council for SR [58]. Feed was provided in individual troughs, and animals had ad libitum access to clean drinking water and mineral blocks.

### 2.3. Experimental Design and Treatments

Bucks were distributed into a randomized complete block design with multiple replicates across time, where each buck served as its own block. The treatments evaluated were (1) OXYT: 10 IU of oxytocin per buck (Oxitocín^®^, Aranda Salud Animal, Querétaro, Querétaro, Mexico), (2) CARB: 0.2 mg 100 kg^−1^ LW^−1^ of carbetocin (Carbeto Jet NRV^®^, Laboratorios Norvet, Monterrey, Nuevo León, Mexico), and (3) CONT: 1 mL of 0.9% sterile saline solution (Solución CS PiSA^®^, PiSA Salud Animal, Guadalajara, Jalisco, Mexico). Hormonal doses were established based on previous studies evaluating these peptides in SR [19,20,22,26]. The saline volume represented the average equivalent volume (i.e., mL) of the hormonal treatments. All treatments were administered intravenously 10 min prior to semen collection. Evaluations were performed bimonthly over one year, totaling six 1-month collection periods. Each evaluation month comprised three collection sessions separated by an 8-day interval, with a 30-day rest period between consecutive evaluation months. Consequently, within each evaluation period, bucks were randomly allocated to a treatment sequence to ensure that all animals received every treatment exactly six times across the entire study. To ensure experimental uniformity, the same veterinarian administered all treatments; because each treatment was administered individually, each buck was considered the experimental unit.

### 2.4. Semen Collection

Semen collection was performed during the morning hours prior to feeding. To simulate natural mating conditions, collections were carried out using an SR-AV (ARBiotech, Culiacán, Sinaloa, Mexico) prepared and operated according to the protocols of Bálcazar-Sánchez and Porras-Almeraya [59]. An estrogenized doe—induced with 2 mg of estradiol benzoate (EstroActive^®^, Virbac, Zapopan, Jalisco, Mexico) intramuscularly—was exposed to all males to serve as a stimulus. During collection, the free end of the AV was positioned at a 45° angle to deflect the buck’s penis into the device [60]. Immediately after ejaculation, the collection tube was detached and transferred to an adjacent covered laboratory located 10 m from the collection site, ensuring that transport time did not affect semen quality. All semen collection procedures were performed by the same trained veterinarian to maintain technical consistency.

### 2.5. Response Variables

Upon arrival at the laboratory, semen samples were immediately placed in a water bath at a constant temperature of 37 °C using a thermostat water bath (model HH2, Civeq^®^, Zhejiang, China). Unless otherwise specified, all microscopic evaluations were performed using a phase-contrast microscope (Nikon, Model Eclipse E200, Nikon, Shanghai, China). The seminal parameters evaluated were as follows:

#### 2.5.1. Volume of the Ejaculate

The VE (mL) was determined by aspirating the total volume of the sample using graduated micropipettes (Gilson, Valle del Oise, France) calibrated to a 1000 μL scale as previously reported [26].

#### 2.5.2. Sperm Concentration

SC (×10^9^ mL^−1^) was assessed using a photometer (model SDM 1, Minitube, Tiefenbach, Germany) previously calibrated for caprines. For this analysis, a droplet of fresh semen was loaded into the slot of a specialized microcuvette and subsequently inserted into the photometer for automated reading [26].

#### 2.5.3. Sperm Mass Motility

Sperm Mass Motility (SMM) (scored on a 0–5 scale) was evaluated by placing a 10 µL aliquot of fresh semen onto a pre-warmed (37 °C) glass slide positioned on a thermal stage and examined under 4× magnification [26]. This response variable was scored based on the motion and wave pattern intensity [59] as follows: 0 = no motion; 1 = minimal or near-absent oscillation; 2 = sporadic oscillation; 3 = generalized oscillation; 4 = slow wave motion; and 5 = rapid, vigorous wave motion.

#### 2.5.4. Total Ejaculated Sperm

The total number of ejaculated spermatozoa (TES) (×10^9^) was calculated mathematically by multiplying the total EV by its respective SC.

#### 2.5.5. Potential Number of Doses for Intravaginal Artificial Insemination

The projected or theoretical number of doses for intravaginal AI (POTNDIAI; n) was calculated based on a standard concentration requirement of 200 × 10^6^ spermatozoa per dose of fresh semen; this value was obtained by dividing the TES by the required insemination dose concentration [61].

#### 2.5.6. Percentage of Viable Sperm

Viable sperm (VS) was assessed using the eosin–nigrosin staining technique. A 10 µL droplet of fresh semen was mixed thoroughly with 10 µL of eosin–nigrosin stain on a glass slide, and a smear was prepared at a 45° angle. The smear was air-dried at room temperature and evaluated under a microscope at 100× magnification (oil immersion). A total of 200 spermatozoa were counted across different fields and classified as stained (i.e., dead) or unstained (i.e., viable); the results are expressed as a percentage of live sperm [62].

#### 2.5.7. Percentage of Sperm with Progressive Motility

To evaluate individual sperm progressive motility (SPM), a 5 µL aliquot of fresh semen was diluted in 1 mL of saline solution inside an Eppendorf tube pre-warmed to 37 °C in a water bath. Then, a 10 µL droplet of this mixture was placed on a pre-warmed slide and covered with a coverslip. The sample was microscopically examined under 40× magnification across multiple fields. This variable was determined subjectively on a scale from 0% to 100%, where a value of 100% was assigned only if all spermatozoa exhibited linear and progressive forward movement [63].

#### 2.5.8. Percentage of Sperm with Normal Morphology

The percentage of spermatozoa with normal morphology (SNM) was determined using the identical eosin–nigrosin smears prepared for viability assessment. A total of 200 spermatozoa were counted and classified into normal or abnormal morphotypes. Abnormal morphology was characterized by the presence of coiled tails, bent tails, detached heads, or deformed heads [20].

#### 2.5.9. Percentage of Sperm with Functional Membrane

The hypo-osmotic swelling test (HOST) was utilized to determine sperm with functional membrane (SFM) [64]. The hypo-osmotic solution consisted of 1.350 g of fructose and 0.735 g of sodium citrate dissolved in 100 mL of tri-distilled water [65]. For the assay, a 20 µL aliquot of fresh semen was incubated with 2 mL of the HOST solution in an Eppendorf tube at 37 °C for one hour. After incubation, a 10 µL sample was placed onto a pre-warmed slide, gently smeared at a 45° angle, and air-dried. The frotis was microscopically examined under 40× magnification, counting 200 spermatozoa classified into either functional cells (coiled or swollen tails) or non-reactive cells (straight tails) [4].

#### 2.5.10. Percentage of Sperm Acrosome Integrity

Sperm acrosome integrity (SAI) was assessed using Coomassie blue staining. The staining solution comprised 0.22% Coomassie blue G-250 (Sigma-Aldrich, St. Louis, MO, USA), 50% methanol, 10% glacial acetic acid, and 40% tri-distilled water [66]. A thin smear was prepared by spreading 10 µL of fresh semen on a glass slide at a 45° angle, air-dried, and fixed by immersion in 4% formaldehyde (Hycel, Zapopan, Jalisco, Mexico) for 15 min. The slides were then stained with the Coomassie blue solution for 5 min, rinsed gently with distilled water, and air-dried. Evaluation was performed microscopically under 100x magnification (i.e., oil immersion) by counting 200 spermatozoa classified as either acrosome-intact or acrosome-damaged based on the presence or absence of staining in the acrosomal region, respectively [67].

#### 2.5.11. Percentage of Sperm with Chromatin Dispersion

Sperm DNA fragmentation was quantified via the sperm chromatin dispersion (SCD) technique as described by Fernández et al. [68]. This halo-based test involved microscopic evaluation of 300 spermatozoa under 40× magnification after applying the specific treatments detailed in the original protocol [68]. Spermatozoa with non-fragmented DNA exhibited a distinct chromatin dispersion halo around the core due to nuclear protein depletion. Halos were classified as large or medium for non-fragmented DNA and small, absent, or degraded for fragmented DNA [68,69]. The final index of spermatozoa with non-fragmented DNA was expressed as a percentage. To ensure procedural uniformity, all semen evaluations were conducted by the same technician in a blinded manner.

### 2.6. Statistical Analyses

Data were analyzed using a Linear Mixed Model (LMM) for repeated measures ANOVA. Treatment, evaluation month, and their interaction (Treatment × Month) were included as fixed factors, while individual animal ID was defined as the random subject factor to account for the dependency of repeated observations within the same buck. Phenotypic correlations between response variables were evaluated using Pearson correlation coefficients. Prior to analysis, normality and homoscedasticity of residuals were verified using Shapiro–Wilk and Levene’s tests, respectively. When fixed effects or interactions were significant (*p* ≤ 0.05), means were compared using Tukey’s post hoc test. Data are presented as mean ± standard error of the mean (S.E.M.). All statistical analyses were performed using Minitab^®^ software (v. 20.4, Minitab LLC, State College, PA, USA).

## 3. Results

### 3.1. Main Effect of Hormonal Treatments upon Semen Characteristics

Hormonal treatments significantly altered all evaluated macroscopic and microscopic semen parameters (*p* ≤ 0.05; Table 1), with CARB eliciting the most pronounced physiological responses. While the overall mean VE was 0.72 mL, CARB administration increased this parameter by 103% compared to both the OXYT and CONT groups. A similar pattern was observed for SC, where the CARB group surpassed the overall mean by 4% (4.60 vs. 4.44 × 10^9^ mL^−1^). Driven by this concurrent expansion of both VE and cellular concentration, TES and the subsequent number of available POTNDIAI experienced a pronounced 116% increase under the CARB treatment compared to the other two experimental groups. Consequently, CARB-treated bucks yielded an average of 13.44 additional POTNDIAI per single ejaculate execution.

Regarding kinetic and structural integrity endpoints, SMM reached its absolute maximum threshold in the CARB group, differing (*p* ≤ 0.05) from CONT, which dropped below the general average. Structural and functional traits followed a matching directional trend, namely VS, SPM, SNM, SFM, and SAI, which consistently established their highest statistical tiers under the long-acting CARB analogue (*p* ≤ 0.05; Table 1). Crucially, OXYT failed to provide any statistical improvement over CONT for SFM and SAI safety markers. Cellular defense was further highlighted by the SCD, which separated the treatments into three distinct statistical hierarchies (*p* ≤ 0.001), demonstrating the highest percentage of spermatozoa with non-fragmented DNA in CARB, followed sequentially by OXYT and CONT. Detailed means and S.E. for all response variables across treatments are presented in Table 1.

### 3.2. Temporal Dynamics and Collection Month Fluctuations in Seminal Quality Parameters

The evaluation across collection months revealed that the majority (64%) of the percentage-based structural variables achieved their highest quantitative means during the initial phase in January (Table 2). Conversely, macro-productive variables such as VE, TES, and POTNDIAI, along with SMM and SAI, remained remarkably stable, displaying no seasonal variations throughout the year (*p* > 0.05). In contrast, specific structural and kinetic markers unmasked distinct time-dependent sensitivities. SC experienced fluctuations across the year (*p* ≤ 0.05), maintaining elevated plateaus during the spring and summer months (May and July) but encountering a sharp depression during early spring in March. Furthermore, SNM fell during the mid-summer heat of July (*p* ≤ 0.05), exhibiting a broad 47% individual range between the absolute poorest performance found in CONT (i.e., 51%) and the maximum structural safety recorded in CARB (i.e., 98%). The most time-sensitive biomarker was the SFM, which plummeted during July and September compared to January (*p* ≤ 0.01). Table 2 details the means and the most conservative S.E. for each response variable across time.

### 3.3. Temporal Micro-Dynamic Treatment by Time Interaction in Seminal Quality Parameters

The interaction between hormonal treatment and experimental period (initial, middle, and final) had a decisive effect (*p* ≤ 0.05) on sperm productivity and cell preservation (Table 3). During the mid-period (July), the CONT group experienced a pronounced physiological decline, marked by sharp reductions in VE and SMM. Notably, pre-collection administration of CARB mitigated this seasonal decline, maintaining high, stable volumes and a perfect SMM score of 5.0 across all evaluated phases. This protective effect was similarly reflected in total output metrics (i.e., TES and POTNDIAI), where untreated CONT males reached a critical production low in July, yielding a minimum average of only 6.6 POTNDIAI per ejaculate. In sharp contrast, CARB stabilized cellular delivery, consistently sustaining high outputs that exceeded 21 doses even during peak summer stress. For qualitative traits, the interaction revealed a severe drop in SPM and SNM in CONT males during July. However, this degradation was fully mitigated by CARB, restoring parameters to baseline control levels (*p* > 0.05) and maintaining both SPM and SNM above 91% during that period. According to Tukey’s post hoc analysis, SC and SAI were the only exceptions, showing no differences among interaction means (*p* > 0.05), with homogeneous performance across all combination tiers. ng a homogeneous performance across all combination tiers.

### 3.4. Phenotypic Co-Dependencies and Matrix Correlation Analysis of Seminal Quality Parameters

**Figure 1:**
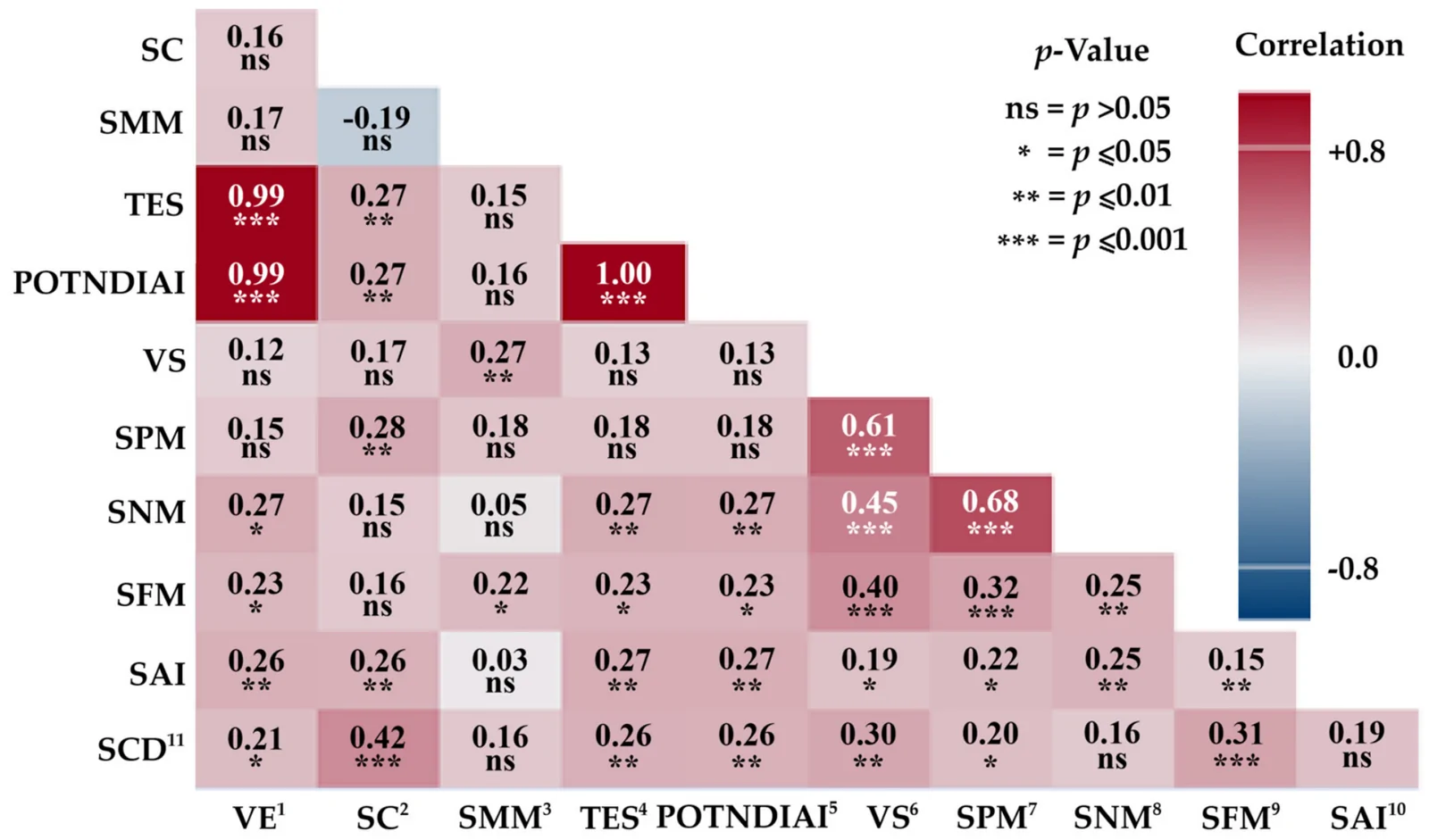
Pearson correlation matrix among the response variables of fresh semen collected using an artificial vagina and analyzed by conventional microscopy in French-Alpine bucks treated with oxytocin (OXI; 10 IU per animal), carbetocin (CAR; 0.2 mg per 100 kg of live weight) or without treatment (CON; 1 mL saline solution per animal) applied 10 min before collection in northern Mexico. ^1^ VE: volume of the ejaculate (mL); ^2^ SC: sperm concentration (×10^9^ mL^−1^); ^3^ SMM: sperm mass motility (0–5); ^4^ TES: total ejaculated sperm (×10^9^); ^5^ POTNDIAI: projected number of doses for intravaginal artificial insemination (n); ^6^ VS: viable sperm (%); ^7^ SPM: sperm with progressive motility (%); ^8^ SNM: sperm with normal morphology (%); ^9^ SFM: sperm with functional membrane (%); ^10^ SAI: sperm with acrosome integrity; ^11^ SCD: sperm with chromatin dispersion (%).

The Pearson correlation matrix exposed the mathematical and biological networks governing the fresh semen architecture of these French-Alpine bucks, highlighting that the volume and concentration expansion induced by OXYT analogues does not compromise cellular integrity (Figure 1). When analyzing the quantitative production axis, VE displayed a nearly perfect positive correlation with both TES (r = 0.99; *p* ≤ 0.01) and POTNDIAI (r = 0.99; *p* ≤ 0.01). Consequently, a perfect linear dependency was confirmed between TES and POTNDIAI (r = 1.0; *p* ≤ 0.01). Interestingly, VE showed slight but significant positive relationships with structural traits such as SNM (r = 0.27; *p* ≤ 0.05), SAI (r = 0.26; *p* ≤ 0.01), and SFM (r = 0.23; *p* ≤ 0.05), while maintaining no association with SC.

On the other hand, SC demonstrated positive correlations with TES (r = 0.27; *p* ≤ 0.01), POTNDIAI (r = 0.27; *p* ≤ 0.01), SPM (r = 0.28; *p* ≤ 0.01), and SAI (r = 0.26; *p* ≤ 0.01). Most notably, SC established its strongest qualitative bond with SCD (r = 0.42; *p* ≤ 0.001). The qualitative kinetic–structural axis revealed that SMM was positively tied to VS (r = 0.27; *p* ≤ 0.01) and SFM (r = 0.22; *p* ≤ 0.05). Additionally, SPM acted as a central indicator of cell health, correlating strongly with viable VS (r = 0.61; *p* ≤ 0.001), SNM (r = 0.68; *p* ≤ 0.001), SFM (r = 0.32; *p* ≤ 0.01), SAI (r = 0.22; *p* ≤ 0.05), and SCD (r = 0.20; *p* ≤ 0.05). Moreover, SFM was confirmed as a vital structural anchor, exhibiting positive associations with VS (r = 0.40; *p* ≤ 0.001), SNM (r = 0.25; *p* ≤ 0.01), SAI (r = 0.15; *p* ≤ 0.01), and SCD (r = 0.31; *p* ≤ 0.001).

## 4. Discussion

**Figure 2:**
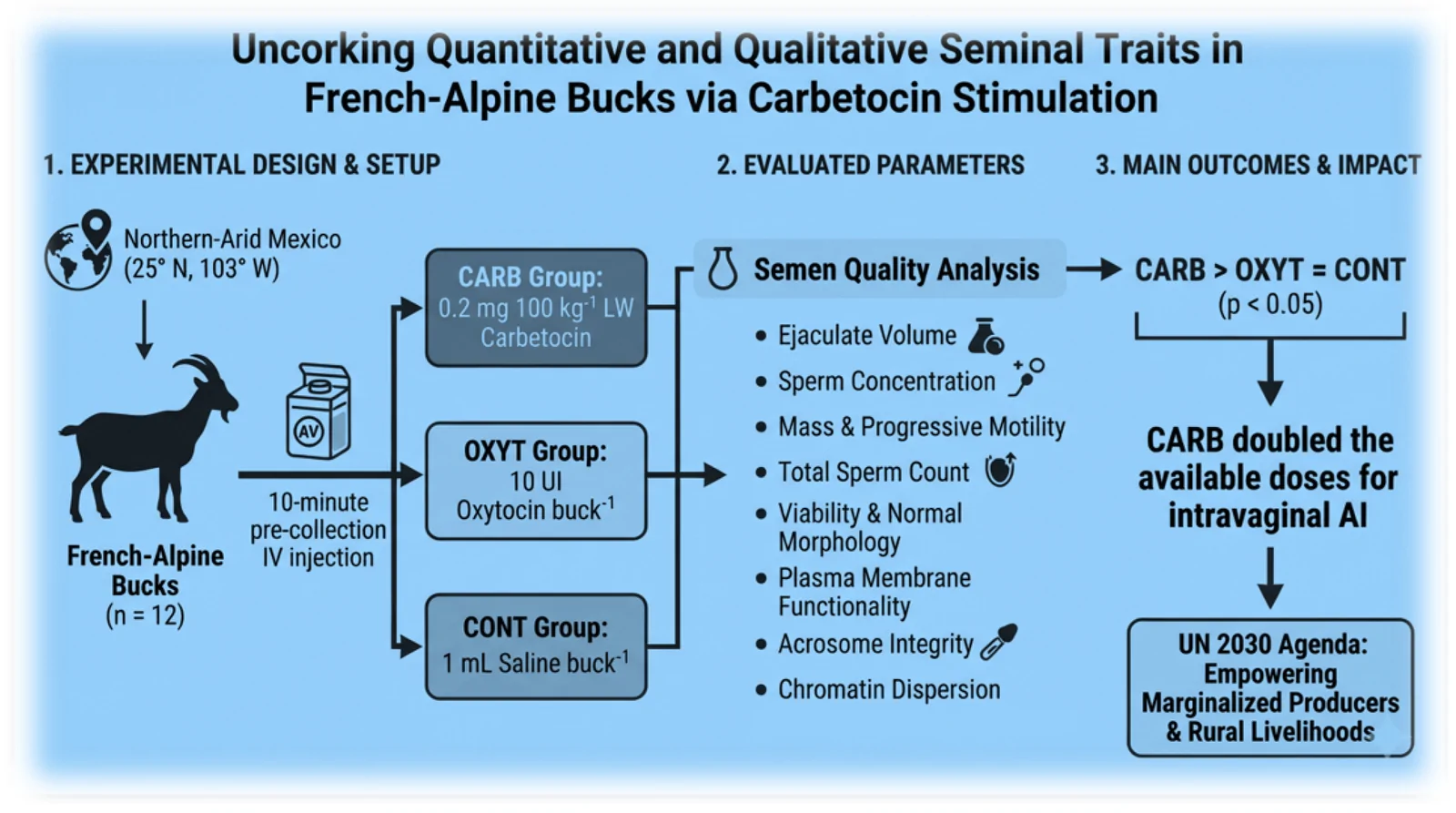
Schematic overview of the longitudinal experimental design and main outcomes in French-Alpine bucks. The timeline illustrates the acute pharmacological intervention 10 min prior to semen collection via artificial vagina (AV), partitioning the experimental units into carbetocin (CARB), oxytocin (OXYT), and saline control (CONT) groups in northern-arid Mexico (25° N, 103° W). Pre-collection CARB stimulation (0.2 mg 100 kg^−1^ LW) via IV significantly outperforms both OXYT and CONT cohorts (*p* ≤ 0.05), eliciting a simultaneous increase in quantitative yields—evidenced by a 103% expansion in volume of the ejaculate, increased sperm concentration, and total sperm count—and qualitative traits, including mass and progressive motility, membrane functionality, acrosome integrity, and diminished chromatin dispersion. This physiological upgrade exerts a compounding effect on reproductive performance, doubling the potential number of doses for intravaginal artificial insemination (A.I.).

The outcomes of this study strongly support our working hypothesis, demonstrating that the intravenous pre-collection administration of CARB significantly outperforms both OXYT and CONT in enhancing the seminal architecture of French-Alpine bucks evaluated via conventional microscopy (Figure 2). Crucially, CARB drove a 103% expansion in VE compared to the other groups. Although intra-epididymal pressures were not directly measured, this simultaneous leap in fluid volume and overall cellular yield likely stems from CARB-induced rhythmic, sustained contractile movements within the cauda epididymidis and accessory sex glands [15,70,71].

### 4.1. Pharmacokinetic Superiority and Metabolic Stability of Carbetocin

By optimizing both SC and total cellular output, CARB effectively doubled the net available commercial POTNDIAI (Table 1). These results closely align with previous SR models where a single pre-collection intervention with this long-acting agonist duplicated the production of POTNDIAI per collection window [26,28]. The clear physiological superiority of CARB over OXYT is fundamentally dictated by their contrasting metabolic stabilities. In SR, native OXYT possesses an extremely fragile plasma half-life restricted to merely two or three minutes, creating a narrow and volatile technical window for sire stimulation [22,24]. Conversely, the synthetic analogue CARB exhibits structural modifications that protect it from rapid enzymatic degradation, maintaining pharmacologically active levels up to ten times longer [26,27,28,46]. This prolonged bioavailability ensures a deep, complete, and highly efficient evacuation of the epididymal sperm reserves [26,27,28,46].

### 4.2. Mechanisms of Spermatic Flow and Comparative Breed Outcomes

Our observations match reports in ovine models, where pre-collection CARB administration boosted total sperm productivity without altering basal cell functionality [26,27,28]. This significant rise in concentration indicates that CARB triggers sustained, coordinated contractions within the myoepithelial cells of the seminiferous tubules and efferent ducts, drastically accelerating spermatic flow into the pelvic urethra [17,21,72,73]. Furthermore, this enhanced bioavailability introduces crucial technical flexibility into routine collection protocols by sustaining its oxytocic effect throughout the entire mating and extraction process [26,27,28,29,30,31]. The quantitative values achieved under CARB stimulation surpassed the baselines reported across various goat breeds in several geographical regions [74,75,76,77,78,79,80,81,82,83,84,85,86,87], although they remained lower than specific highly prolific profiles [88,89,90]. These cross-study variations in VE and cellular concentration are expected, as they are typically driven by complex interactions between breed, age, body condition, nutrition, collection frequencies, macroclimatic variations, and the execution of false mounts prior to collection. The quantitative outputs generated by the OXYT and CONT cohorts were inferior to previous reports [74,75,76,77,78,79,80,81,82,83,84,85,86,87,88,89,90].

### 4.3. Quantitative Semen Trait Improvement and Membrane Protection

Regarding qualitative parameters, CARB consistently increased the percentages of SPM, SNM, and SFM spermatozoa across the experimental timeline (Table 1 and Table 2). According to the phenotypic correlation analysis, plasma membrane integrity—quantified via the HOST—was positively associated with SC (Figure 1). This relationship suggests that CARB stimulation does not merely increase seminal volume through simple fluid dilution; rather, it optimizes the biochemical microenvironment of the seminal plasma, shielding the spermatozoa from mechanical shear stress during the friction of ejaculation [10,26]. While OXYT administration can transiently improve motility in ruminants, its practical field effects remain highly inconsistent due to its rapid clearance [18,19,20,91]. Prolonged and steady agonist stimulation of the accessory sex gland receptors may alter the protein and metabolite composition of the seminal fluid, adding a biochemical layer of protection to the plasmalemma [10,26]. Consequently, as a long-acting agonist, CARB guarantees persistent activation of the reproductive tract OXYT receptors, translating directly into superior structural and genomic quality [26,27,92].

### 4.4. Discrepancies, Variable Correlations, and Seasonal Adaptability

In contrast, the observed significant treatment effects on percentage-based structural traits differ from several fresh semen studies across alternative caprine genotypes where hormonal aids failed to modify cell quality [63,75,77,79,80,82,85,86,87,88,89,93,94,95]. These discrepancies are likely modulated by individual variations in semen osmolality, baseline pH, nutritional planes, and strict ambient temperatures across reference centers [77]. The high mathematical interdependence detected among our quantitative variables was biologically expected. For instance, the robust positive correlation between SMM and SPM accurately reflects the synchronized kinetic competence of individual spermatozoa both in bucks [77,79,87,88,96,97] and bulls [98]. Finally, the high stability of most seminal parameters across months confirms the remarkable climatic adaptability of the French-Alpine breed to the semiarid conditions of northern Mexico (Table 2). Although absolute averages peaked during the optimal winter conditions of January, CARB successfully maintained its technical efficacy regardless of the environmental phase. This was most compellingly demonstrated during the mid-summer heat of July, where CARB was the single treatment capable of sustaining superior motility and VE, while the untreated CONT group plummeted to production lows (Table 3).

## 5. Conclusions

While both CARB and OXYT enhance caprine seminal parameters, CARB outperformed both OXYT and CONT groups in VE, SC, total sperm count, and projected POTNDIAI in French-Alpine bucks in northern-arid Mexico. Furthermore, CARB improved SPM, plasma membrane integrity, and SNM without compromising cellular structure or function. The persistent efficacy of this synthetic analogue throughout all seasonal dynamics positions it as a superior alternative for optimizing semen collection, AI programs, and genetic preservation. Consequently, integrating CARB into commercial buck-selection centers and smallholder farms—particularly in marginalized regions where accessible reproductive technologies directly safeguard rural livelihoods—emerges as a valid reproductive management strategy. Future research should evaluate its impact on in vivo fertility, cryopreservation survival, and reproductive performance across diverse seasonal and management conditions. Ultimately, by bridging advanced pharmacology with practical caprine production, CARB transforms routine semen collection into a highly efficient biotechnological tool to enhance production outcomes, thereby driving socioeconomic resilience and sustainable development for smallholder livestock producers.

## Figures and Tables

**Table 1:** Mean ± standard error of the mean per treatment for the response variables of fresh semen collected using an artificial vagina and analyzed by conventional microscopy in French-Alpine bucks (n = 12) treated with oxytocin (OXYT; 10 IU per animal), carbetocin (CARB; 0.2 mg per 100 kg LW), or no treatment (CONT; 1 mL saline solution per animal) applied 10 min before collection in northern Mexico (25° N).

Variable ^1^/Treatment	OXYT	CARB	CONT	S.E.M. ^2^	*p*-Value
VE (mL)	0.53 ^b^	1.09 ^a^	0.54 ^b^	0.07	0.001
SC (×10^9^ mL^−1^)	4.42 ^b^	4.59 ^a^	4.30 ^b^	0.05	0.002
SMM (0–5)	4.81 ^a,b^	5.00 ^a^	4.53 ^b^	0.17	0.008
TES (×10^9^)	2.33 ^b^	5.02 ^a^	2.32 ^b^	0.03	0.001
POTNDIAI (n)	11.14 ^b^	24.58 ^a^	11.14 ^b^	1.59	0.001
VS (%)	82.81 ^a^	84.86 ^a^	78.75 ^b^	0.99	0.001
SPM (%)	87.36 ^a,b^	90.00 ^a^	85.14 ^b^	1.46	0.047
SNM (%)	90.83 ^a^	93.17 ^a^	88.08 ^b^	0.79	0.001
SFM (%)	77.86 ^b^	82.86 ^a^	74.33 ^b^	1.37	0.001
SAI (%)	90.06 ^b^	93.36 ^a^	89.58 ^b^	0.81	0.001
SCD (%)	95.61 ^b^	97.44 ^a^	94.33 ^c^	0.37	0.001

^1^ Response variables: VE: volume of the ejaculate; SC: sperm concentration; SMM: sperm mass motility; TES: total ejaculated sperm; POTNDIAI: potential number of doses for intravaginal artificial insemination; VS: viable sperm; SPM: sperm with progressive motility; SNM: sperm with normal morphology; SFM: sperm with functional membrane; SAI: sperm with acrosome integrity; SCD: sperm with chromatin dispersion; ^2^ S.E.M.: the most conservative standard error of the mean is presented; ^a–c^ means with different superscripts within the same variable differ among periods (months) (*p* ≤ 0.05).

**Table 2:** Mean ± standard error of the mean across time for diverse response variables of fresh semen collected using an artificial vagina and analyzed by conventional microscopy in French-Alpine bucks (n = 12) treated with oxytocin (OXYT; 10 IU per animal), carbetocin (CARB; 0.2 mg per 100 kg LW), or no treatment (CONT; 1 mL saline solution per animal) applied 10 min before collection in northern Mexico (25° N).

Variables ^1^/Month	Jan	Mar	May	Jul	Sep	Nov	S.E.M. ^2^	*p*-Value
VE (mL)	0.87 ^a^	0.68 ^a^	0.70 ^a^	0.56 ^a^	0.82 ^a^	0.72 ^a^	0.08	0.287
SC (×10^9^ mL^−1^)	4.36 ^a,b^	4.28 ^b^	4.59 ^a^	4.59 ^a^	4.41 ^a,b^	4.38 ^a,b^	0.05	0.044
SMM (0–5)	4.94 ^a^	4.94 ^a^	4.72 ^a^	4.44 ^a^	4.78 ^a^	4.83 ^a^	0.06	0.210
TES (×10^9^)	3.79 ^a^	2.91 ^a^	3.26 ^a^	2.57 ^a^	3.64 ^a^	3.18 ^a^	0.03	0.436
POTNDIAI (n)	18.44 ^a^	14.00 ^a^	15.78 ^a^	12.39 ^a^	17.67 ^a^	15.44 ^a^	1.72	0.438
VS (%)	83.67 ^a^	83.89 ^a^	82.33 ^a^	79.17 ^a^	83.06 ^a^	80.72 ^a^	0.55	0.087
SPM (%)	89.72 ^a^	88.61 ^a^	87.22 ^a^	82.50 ^a^	88.89 ^a^	88.06 ^a^	0.28	0.124
SNM (%)	91.33 ^a^	90.72 ^a^	90.72 ^a^	85.83 ^b^	92.44 ^a^	93.11 ^a^	0.80	0.004
SFM (%)	83.94 ^a^	80.22 ^a^	80.28 ^a^	72.39 ^b^	73.67 ^b^	76.61 ^a^	1.21	0.001
SAI (%)	90.56 ^a^	91.17 ^a^	91.39 ^a^	91.67 ^a^	92.22 ^a^	89.00 ^a^	1.11	0.394
SCD (%)	95.33 ^a^	94.22 ^a^	95.78 ^a^	96.39 ^a^	95.78 ^a^	96.28 ^a^	0.40	0.711

^1^ Response variables: VE: volume of the ejaculate; SC: sperm concentration; SMM: sperm mass motility; TES: total ejaculated sperm; POTNDIAI: potential number of doses for intravaginal artificial insemination; VS: viable sperm; SPM: sperm with progressive motility; SNM: sperm with normal morphology; SFM: sperm with functional membrane; SAI: sperm with acrosome integrity; SCD: sperm with chromatin dispersion; ^2^ S.E.M.: the most conservative standard error of the mean is presented; ^a,b^ means with different superscripts within the same variable differ among periods (months) (*p* ≤ 0.05).

**Table 3:** Mean ± standard error of the mean of the treatment by time interaction for the response variables of fresh semen collected using an artificial vagina and analyzed by conventional microscopy in French-Alpine bucks (n = 12) treated with oxytocin (OXYT; 10 IU per animal), carbetocin (CARB; 0.2 mg per 100 kg LW), or no treatment (CONT; 1 mL saline solution per animal) applied 10 min before collection in northern Mexico (25° N).

Variables ^1^ & Periods	Treatment									S.E.M. ^5^
	OXYT			CARB			CONT			
	Initial ^2^	Middle ^3^	Final ^4^	Initial	Middle	Final	Initial	Middle	Final	
VE (mL)	0.72 ^a–d^	0.43 ^c,d^	0.55 ^b–d^	1.20 ^a,b^	0.92 ^a–d^	1.03 ^a–c^	0.68 ^a–d^	0.32 ^c,d^	0.57 ^b–d^	0.05
SC (×10^9^ mL^−1^)	4.28 ^a^	4.62 ^a^	4.37 ^a^	4.49 ^a^	4.69 ^a^	4.60 ^a^	4.32 ^a^	4.46 ^a^	4.20 ^a^	0.03
SMM (0–5)	4.83 ^a^	4.83 ^a^	4.83 ^a^	5.00 ^a^	5.00 ^a^	5.00 ^a^	5.00 ^a^	3.50 ^b^	4.67 ^a,b^	0.10
TES (×10^9^)	3.10 ^a–e^	1.90 ^d,e^	2.37 ^b–e^	5.37 ^a,b^	4.39 ^a–e^	4.75 ^a–d^	2.91 ^a–e^	1.43 ^e^	2.42 ^b–e^	0.02
POTNDIAI (n)	15.00 ^a–e^	9.00 ^d,e^	11.50 ^b–e^	26.33 ^a,b^	21.50 ^a–e^	23.17 ^a–d^	14.00 ^a–e^	6.67 ^e^	11.67 ^b–e^	1.31
VS (%)	84.50 ^a,b^	78.33 ^a–c^	83.00 ^a–c^	84.33 ^a,b^	85.17 ^a,b^	83.17 ^a–c^	82.17 ^a–c^	74.00 ^c^	76.00 ^b,c^	0.56
SPM (%)	89.17 ^a,b^	82.50 ^a,b^	88.30 ^a,b^	90.00 ^a^	91.67 ^a^	90.00 ^a^	90.00 ^a^	73.33 ^b^	85.83 ^a,b^	0.95
SNM (%)	91.00 ^a,b^	85.50 ^a,b^	94.50 ^a^	92.17 ^a^	91.17 ^a,b^	96.17 ^a^	90.83 ^a,b^	80.83 ^b^	88.67 ^a,b^	0.70
SFM (%)	83.67 ^a^	72.17 ^a–c^	78.67 ^a–c^	87.17 ^a–c^	77.83 ^a–c^	84.83 ^a^	81.00 ^a-c^	67.17 ^b,c^	75.33 ^a–c^	0.92
SAI (%)	91.17 ^a^	92.83 ^a^	86.67 ^a^	92.83 ^a^	92.50 ^a^	93.67 ^a^	87.67 ^a^	89.67 ^a^	86.67 ^a^	0.22
SCD (%)	95.83 ^a,b^	95.67 ^a,b^	96.00 ^a,b^	97.00 ^a,b^	99.00 ^a^	97.33 ^a,b^	93.17 ^b^	94.50 ^a,b^	95.50 ^a,b^	0.32

^1^ Response variables: VE: volume of the ejaculate; SC: sperm concentration; SMM: sperm mass motility; TES: total ejaculated sperm; POTNDIAI: potential number of doses for intravaginal artificial insemination; VS: viable sperm; SPM: sperm with progressive motility; SNM: sperm with normal morphology; SFM: sperm with functional membrane; SAI: sperm with acrosome integrity; SCD: sperm with chromatin dispersion. ^2^ Initial corresponds to January. ^3^ Middle corresponds to July. ^4^ Final corresponds to November. ^5^ S.E.M.: The most conservative standard error of the mean is presented. ^a–e^ Means with different superscripts within the same variable differ for the treatment and period interaction (*p* ≤ 0.05).

## Data Availability

The datasets used in this research are available from the corresponding author upon reasonable request.

## Abbreviations

The following abbreviations are used in this manuscript:

AI	Artificial insemination
AV	Artificial vagina
CARB	Carbetocin
CASA	Computer-assisted semen analysis
CONT	Control group (no treatment)
HOST	Hypo-osmotic swelling test
OXYT	Oxytocin
SAI	Sperm with acrosome integrity
SC	Sperm concentration
SCD	Sperm with chromatin dispersion
S.E.M.	Standard error of the mean
SFM	Sperm with functional membrane
SMM	Sperm mass motility
SNM	Sperm with normal morphology
SPM	Sperm with progressive motility
SR	Small ruminant
TES	Total ejaculated sperm
POTNDIAI	Potential number of doses for intravaginal artificial insemination
VE	Volume of the ejaculate
VS	Viable sperm
